# A Review of the Use of Linear Programming to Optimize Diets, Nutritiously, Economically and Environmentally

**DOI:** 10.3389/fnut.2018.00048

**Published:** 2018-06-21

**Authors:** Corné van Dooren

**Affiliations:** Voedingscentrum, The Netherlands Nutrition Centre, Den Haag, Netherlands

**Keywords:** sustainable diet, linear programming, diet costs, nutritional quality, environmental constraints (EC)

## Abstract

The “Diet Problem” (the search of a low-cost diet that would meet the nutritional needs of a US Army soldier) is characterized by a long history, whereas most solutions for comparable diet problems were developed in 2000 or later, during which computers with large calculation capacities became widely available and linear programming (LP) tools were developed. Based on the selected literature (52 papers), LP can be applied to a variety of diet problems, from food aid, national food programmes, and dietary guidelines to individual issues. This review describes the developments in the search for constraints. After nutritional constraints, costs constraints, acceptability constraints and ecological constraints were introduced. The 12 studies that apply ecological constraints were analyzed and compared in detail. Most studies have used nutritional constraints and cost constraints in the analysis of dietary problems and solutions, but such research begin showing weaknesses under situations featuring a small number of food items and/or nutritional constraints. Introducing acceptability constraints is recommended, but no study has provided the ultimate solution to calculating acceptability. Future possibilities lie in finding LP solutions for diets by combining nutritional, costs, ecological and acceptability constraints. LP is an important tool for environmental optimization and shows considerable potential as an instrument for finding solutions to a variety of very complex diet problems.

## Introduction: the diet problem

### Aim

Nutrition is affected by numerous environmental and societal causes. Although the diet problems were already urgent during World War II, the challenge of feeding the world in a healthy and sustainable manner will only become more urgent (1). Herforth et al. (2) proposed a “simple framework based on three domains: nutritional quality, economic viability, and environmental sustainability”. This paper answered their proposal by including the three domains in an integrated way (2). It is expected that LP makes it possible to model these domains across disciplines.

This paper reviews the application of linear programming to optimize diets with nutritional, economic, and environmental constraints. There are three main reasons for studying the application of LP to diets in greater depth:

- Linear programming is thought to be “the ideal tool to rigorously convert precise nutrient constraints into food combinations” (3).
- Maillot et al. (4) stated that most food-based dietary guidelines assume that people eating according guidelines are receiving all recommended nutrients. However, in practice this is not always true (4). So, LP could be helpful to support development of dietary guidelines that fulfill all nutritional requirements.
- Macdiarmid (5) observed that healthy diets have not always lower environmental impacts. She assumed that LP is able to suggest diets and products with lower environmental impacts than the impacts of diets assessed through scenario type studies (5).

The goal of this review is to analyse if the application of LP since 2000 provided acceptable diet solutions in practice, especially when environmental constraints were introduced.

### Definition

Linear Programming (LP) can be used to solve questions on matching diets to nutritional and other additional constraints with a minimum amount of changes. Linear programming is a mathematical technique that allows the generation of optimal solutions that satisfy several constraints at once (6).

### History

The first studies applying LP to diets were published between 1950 and 1960 (7). The search for diet solutions started with Jerry Cornfield, who formulated “The Diet Problem” for the Army during World War II (1941–1945), in search of a low-cost diet that would meet the nutritional needs of a soldier. The economist George Stigler, endeavored optimization techniques to establish the cheapest diet delivering enough energy, proteins, vitamins, and minerals (8). According to Buttriss et al., this diet should be composed by the available list of 77 US foods of which the costs and nutrient composition were measured: “Stigler could not find the exact solution to this problem, which turned out to be incredibly complex. The Stigler “Diet Problem” is a typical question of resource optimization or, in mathematical terms, of minimization of a linear function subject to multiple linear constraints, also called linear programming” (9).

For the duration of World War II, the Air Force and other parts of the army were hiring mathematicians to solve the important diet problem and to plan affordable meals. Among the researchers involved in solving this problem was George Dantzig. He proposed a new algorithm he had developed. It took him until 1947, being the first to deliver the correct mathematical result (9, 10). Dantzig tested his model on his own diet, constructing a database with 50 foods. He wanted to reduce his caloric intake to 1,500 kcal and programmed an objective function to maximize the feeling of being full (operationalized as the weight per unit minus the weight of its water content). The solution he found was a weird diet with 200 bouillon cubes per day. This was possible because the former nutritional requirements didn't show a limit to the amount of salt. These results led to upper bounds being added to LP for the first time (10). Until now the approach has been used in many ways to design individual diets as well as population diets (4). The problem of the diet is interesting, because it is difficult to optimize the function of phenomenon like the diet, as it is composed of several variables: energy density, water content, macronutrients, micronutrients, bioactive substances, and contaminants. This paper gives an overview of those applications.

### Calculation methods

This review focus on optimization through the application of linear programming. This section explains the background of this method. The result of a LP problem shrinks to discover the optimum worth (maximum or minimum, liable to the problem) of the linear equation (named the “objective function”):

$$\begin{array}{l} f=c_{1}x_{1}+\ldots +c_{n}x_{n} \end{array}$$

**Figure 1:**
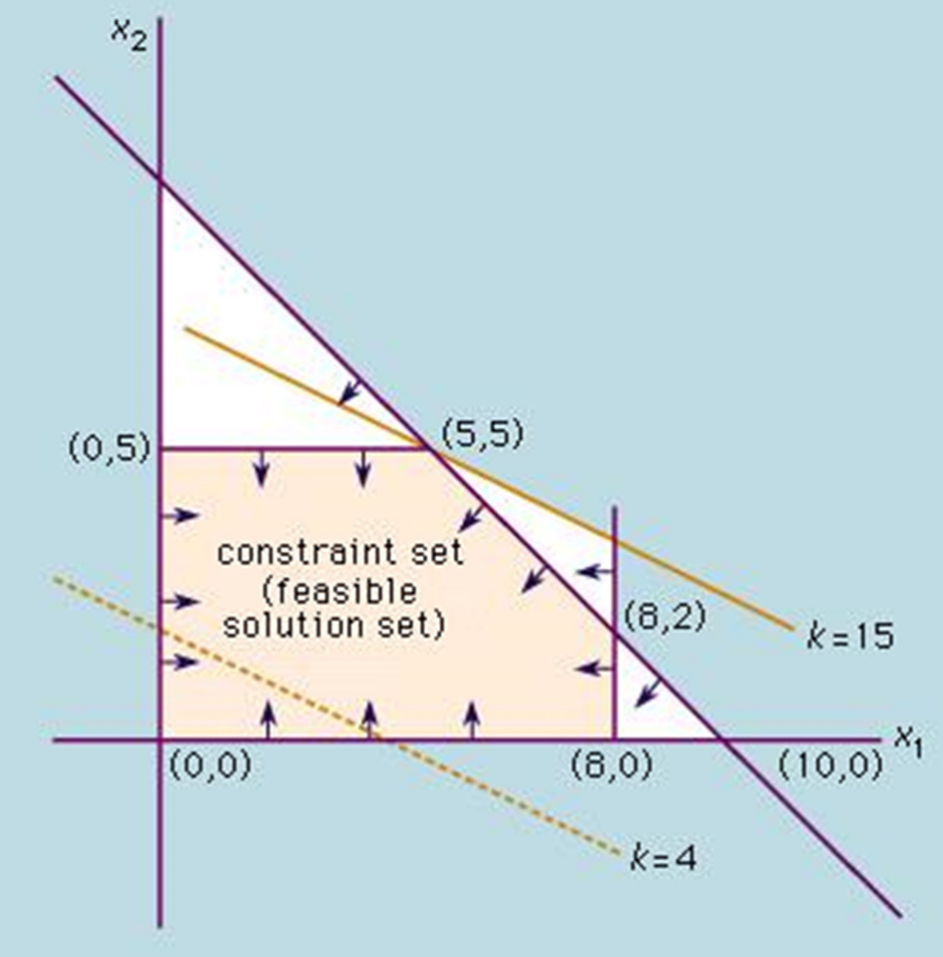
Concept of linear programming: The constraints (x_i_; purple lines) result in a feasible solution set (yellow area). The objective function (k; yellow line) results in the highest possible solution at the edge of the solution area.

The function is conditional on different constraints, stated as inequalities (see Figure 1). According mathematicians “the basic assumption in this method is that the various relationships between demand and availability are linear.” To obtain the solution, “it is necessary to find the solution of the system of linear inequalities (that is, the set of *n*-values of the variables x_i_ that simultaneously satisfies all the inequalities). The objective function is then evaluated by substituting the values of x_i_ in the equation that defines *f* “(Encyclopedia Britannica, accessed June 2nd, 2017).

Mathematician Cornfield started to find solutions for the Diet Problem by constructing an Input-Output model. His colleague Hoffenberg suggested using the simplex method (a standard method of maximizing a linear function of several variables). In 1947, a team took 120-man days to find a solution using the simplex method. A few years later, Dantzig introduced a linear program and started using an IBM 701 computer in the early 1950s (10). The development of diet solutions was highly dependent on the development of computers with a high calculation capacity. The laborious computations necessary for LP were only possible at the time when fast computer technologies became available (3).

Solving such complex problem subjected to several constraints either to optimize the daily energy allowance, macro and micronutrient intakes, or constraints on economic issues (price, income) and environmental issues (greenhouse gas emissions, energy use, land use, exposure to contaminants). Authors assume that constraints, such as price and nutrient content are linearly related to food weight (11), but this could be a simplification of the reality. Micronutrients (e.g., advised daily intake vs. toxicity of Iodine) or costs (e.g., price elasticity) could be non-linear. There are several open problems in the theory of linear programming, for instance the strongly polynomial-time performance in the number of constraints and the number of variables. Besides linear optimization functions, several authors suggest using quadratic functions for optimization on popularity or acceptability (12–14).

## Materials and methods

### Literature review

**Figure 2:**
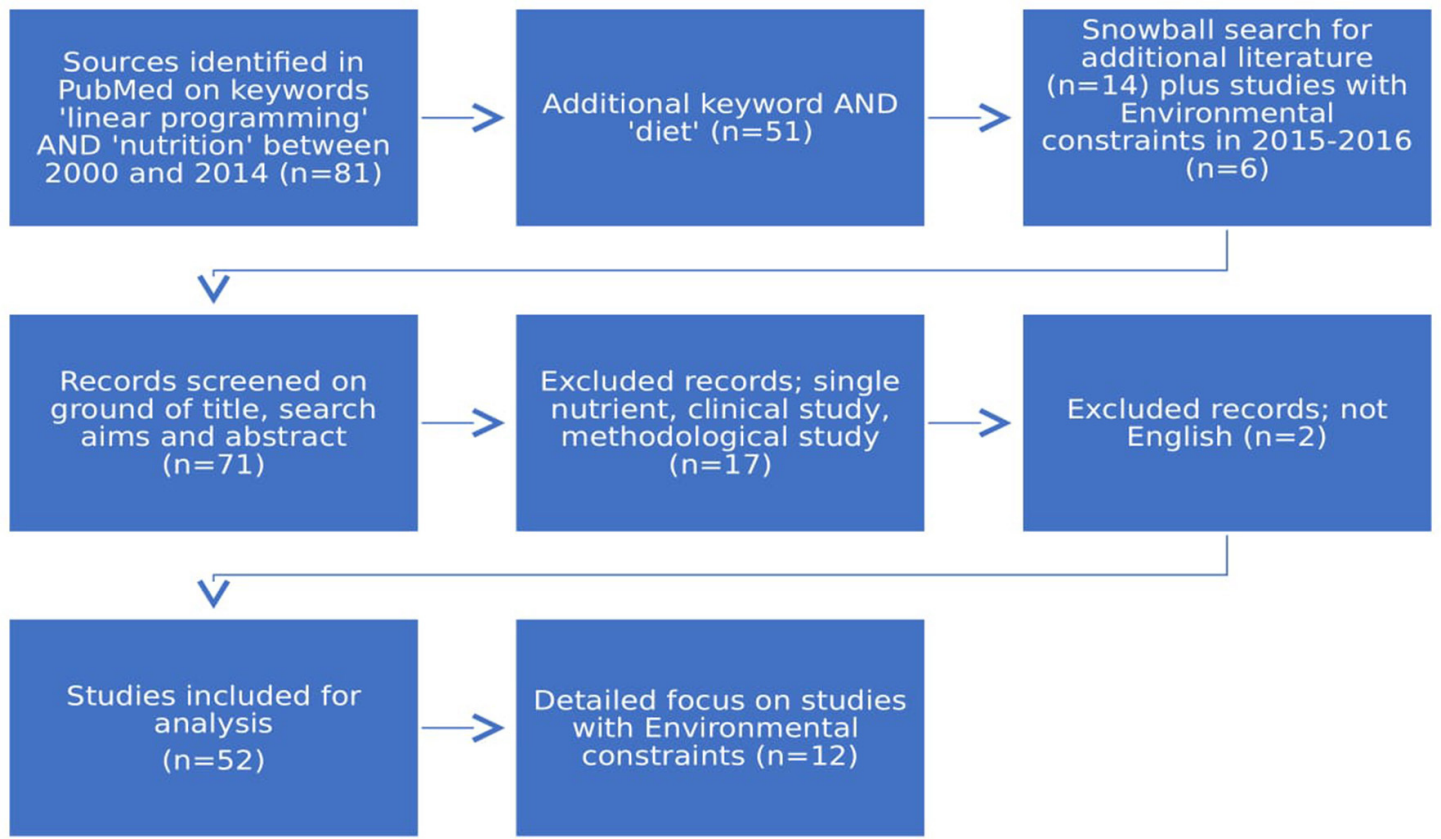
Selection of papers through the PRISMA protocol for systematic literature research.

In this systematic literature review we selected—in line with the PRISMA protocol (Figure 2)—literature on Pubmed: full text articles with “linear programming” and “nutrition” in the title or as key word, published between 2000 and 2014, including review papers (*n* = 81). The selection was narrowed by adding “diet” as key word (*n* = 51). We included through the snowball search approach additional related citations from these articles and from Mertens at al.'s review article (15), and additional studies using environmental constraints published in 2015–2016, resulting in a total of *n* = 71. The records were screened on ground of title, aims and abstract. This resulted in the exclusion of 19 papers, based on the following criteria: non-English language, single nutrient, clinical study, or methodological paper. The total number of studies included for analysis are 52. A short overview of the studies is given in Appendix A. Since the time of “The Diet Problem,” LP has been applied in different sciences, but until 2003 it was rarely applied to questions of human nutrition (3). This review describes the historical developments and improvements of the application of linear programming (LP) in diets since 2000. Five studies applying quadratic programming (QP) are also considered. Detailed focus was on diet studies with ecological constraints (*n* = 12). An extraction table (Table 1)

**Table 1:** Overview of the 12 diet studies with both nutritional and ecological constraints.

**Study**	**Goal**	**Outcome**	**Comment**
(13)	To assess the impact of diet change on the blue and green water footprints of food consumption	Green water: −6, −11, −15, −21%. Blue water: −4, −6, −9, −14%. Halving animal protein saves water for the diet of an additional 1.8 billion people	Recommended diet per country not specified
(16)	Estimate likely changes in diet under healthy eating guidelines and their consequences for the agricultural sector	Increase of 131.4% in gross margins; increase land use of oats, potatoes, fruits, and vegs; decrease use of sugar beet, milk, beef, sheep, beans, and some cereals	
(17)	Whether a reduction in GHGEs can be achieved while meeting dietary requirements	2.43 kg CO_2_eq/d (−36%) and GBP 29.-/wk	No drinks included
(18) (Spain)	To determine whether it is possible to develop corresponding diet recommendations in other countries; to analyse the difficulties of integrating data from multiple sources	25% reduction in GHGe: 2,710g CO_2_e/day. Costs € 3.48 (unchanged)	Ignored the effect of alcohol and drinks
(18) (Sweden)		25% reduction in GHGe: 4,295g CO_2_e/day. Costs SEK 44.07 (−0.57)	All diets show reduction in total amount of meat and increase in legumes and bread/ pasta/ potatoes
(18) (France)		25% reduction in GHGe: 2,609g CO_2_e/day. Costs € 4.36 (−0.54)	
(19)	Ensuring food security in the context of rising food prices and environmental constraints	5.98 kg CO_2_eq/d and NZ$ 6.75	No drinks included
(20)	To find low climate impact diets that are affordable yet fulfill all nutritional requirements	1.58 kg CO_2_eq/day and € 2.57	
(21)	Demonstrate a method that is able to identify diets with reduced environmental impact and that are more similar to the current diet than predetermined scenarios	30% less environmental impact (0.29 pt pReCiPe)	Diet compared with (pesco)vegetarian, vegan, closest healthy
(22)	To model the specific reductions in food-related GHGEs that could be achieved while meeting international dietary recommendations and minimizing deviation from the current diet	WHO guidelines −17% GHGE, realistic modifications −40% GHGE (fewer animal products and processed snacks, more fruit, vegetables, and cereals)	More than 40% is unlikely without radical change
(23)	To assess the compatibility between reduction of diet-related GHGEs and nutritional adequacy, acceptability and affordability dimensions	GHGE reductions up to 30%; higher GHGE reductions decreased diet cost but also diet quality with major shifts in diet	3 levels of nutritional constraints; stepwise 10% GHGE reduction; aggregation into food groups with new Euclidean distance method
(24)	To investigate the diversity in dietary changes needed to achieve a healthy diet and a healthy diet with lower GHGEs by taking into account each individual's current diet and then minimizing the changes they need to make	Only 7.5% of people achieved healthy diet and 4.6% sustainable diet; 15 and 27% reduction in GHGEs, respectively; healthy diets alone do not produce substantial reductions in GHGEs	4 step model; using 7–10 new items, 95% met health or GHGE constraints; sodium most difficult nutrient to meet; healthy diets alone do not produce substantial reductions in GHGE
(25)	To identify a healthy, greener and cheaper diet based on current consumption patterns	More than 50% CO2 reduction for 3 diets to 8.3 kg CO_2_/wk; 10 euro/wk cost reduction (25%) for the low cost diet	
(26)	To demonstrate that linear programming can be used to define nutritionally healthy, environmentally friendly, and culturally acceptable diets, using the Low Lands as an example	Optimized Low Lands Diet results in a lower environmental impact than the Mediterranean and New Nordic Diet; GHGEs are 2.60 kg CO_2_eq/day and LU 2.86 m^2^*year/day	Retrospective study about optimizing the traditional Low Lands Diet

*The table gives details about the goal, outcomes and comments*.

was constructed, including study aim, main outcome, objective function, used programme, applied nutritional, economic and ecological constraints, number of food items included, and study population (country, age, gender).

### Computer programs

Among the LP pioneers were Soden and Fletcher from the University of Salford UK. Their principles of LP were already clear in 1992 and still in use. They were far ahead of their time by using an objective function based on individual food preferences. They developed a computer program named “Microdiet System, 1990” in collaboration with practicing dieticians, which was used in some leading UK hospitals (27). Guided by the philosophy of goal programming, they described a “computational method for constructing individually acceptable diets by modifying a chosen diet to meet nutritional requirements.” They demonstrated the effects of imposing different nutrient requirements (*n* = 4) on small food quantities (*n* = 25) on a sample diet and described techniques which can ensure that the modified diet will be acceptable to the individual. The starting point in the calculation was the person's current dietary intake. This was modified using LP methods which use vectors to make the smallest changes to the food quantities to meet specific targets. Sequential modification was introduced to identify changes that are acceptable to the individual (27). Their maximum capacity was a problem with 100 foods and 30 constraints. Based on this analysis, Fletcher et al. developed a computational method able to construct individually acceptable diets by means of LP (28).

The advent of powerful personal computers made an LP function accessible in widespread computer programmes, for example Microsoft Excel®. Also other spreadsheet programmes now provide a simple solver function that can be used for LP (11). Briend et al. (3) described in detail how to apply this function in Excel. An LP module was also incorporated in diet analysis programs, of which “Nutrisurvey” is a free example (http://www.nutrisurvey.de/lp/lp.htm) (3).

Since 1975, a computerized programming model with a quadratic mathematical function has been available as part of the Thrifty Food Plan (TFP) in the USA (29). The group of Lino designed a Microsoft Excel application in 2008, which permits “one to more easily evaluate the official USDA food plans or to create a new benchmark food plan that meets one's own chosen nutrition policy goals” (30). Gao et al. were among the first to apply Quadratic Programming (QP) in diets. They used SAS (Version 8.02) for the calculations, and exported the data to Microsoft Excel. QP used Microsoft Excel SOLVER developed by Frontline Systems (31). The European HELENA study also applied QP to optimize diets from Food Frequency Questionnaires. The solutions were acquired through the use of LINGO Hyper (Release 10.0, LINDO Systems Inc., USA) (32).

LP and QP are also available in the statistical software R 2012 through a GNU Linear Programming Kit implemented in the IpSolveAPI package (or “Rglpk”). Macdiarmid in the UK was one of the first to use the software for LP in diets for the calculations of WWF Livewell Plate (17, 33). Macdiarmid states that “this mathematical method optimizes an outcome which is a linear function of several variables that can be controlled (e.g., the amount of food eaten), while subject to a number of constraints (e.g., dietary requirements)” (33).

Blonk Consultants in the Netherlands developed their own software Optimeal®, which was used in the LP studies of Van Dooren et al. (20, 26). The tool supports LP and QP and has several options to customize the goal function, such as a proxy of popularity and different measures for distance. Optimeal is programmed in Matlab Compiler 7.16.

## Results

### Nutritional constraints: from food plans to dietary guidelines

This review includes 52 optimization studies with nutritional constraints, of which 17 without other constraints.

At the start of this millennium, the French group lead by Nicole Darmon, Elaine Ferguson (New Zealand), and André Briend started to apply LP in food aid. They demonstrated in Malawi that it was possible to satisfy nutritional recommendations for children 3–6 y during the harvest season using LP through the Microdiet software with a small distance from the native diet. But, in the non-harvest period, the availability of riboflavin and zinc was improved (34). Later, Ferguson and her team (35) were active in Indonesia, applying LP to develop complementary feeding recommendations to apply in specific populations with a diet with micronutrient deficiencies (35).

Inspired by the results of Darmon, Ferguson, and Briend, the UN World Food Programme and Save the Children started applying LP, a few years later. A user-friendly Excel solver function was developed for practitioners by Save the Children UK. With this spreadsheet, LP has been used in many developing countries to assess to what extent economic constraints contribute to the nutrition problems. For this purpose, the organizations have gathered prices of food products from a couple of countries (i.e., Zambia, Mozambique, Djibouti, Bangladesh, Tanzania, and Niger). They called it the “Cost of the Diet” tool. In the tool, the following data were included:

- World Food Composition Database,
- recommended nutrient requirements (WHO data for different ages, gender, and physiological conditions), and
- food prices per 100 g per country.

Romeo Frega et al. published a case study applying the tool in Mozambique to determine cost-effective fortification strategies (36).

In the meantime, the French group worked together with Adam Drewnowski from the University of Washington to apply LP in the USA. The French study applied LP to create food plans that meets critical dietary recommendations advised by the World Cancer Research Fund (37). Consumption constraints were included to prevent food plans from advising unreasonable amounts of food from a food group (Much more stringent than the USDA TFP). Consumption data were taken from a 161 representative men and women in the Pacific Northwest. For this group, achieving cancer prevention recommendations required little modification of their current diets (lower in refined grains and higher in vegetables and fruits) and had small impacts on the cost and quality of the diet. However, to meet all nutritional needs it was necessary to highly increase the volume and change the food intake patterns. Costs were considered, but not as a constraint (diet costs rose from $ 6.95/day to $ 8.03/day for women). These applications demonstrated that optimization models deliver an sophisticated mathematical solution to check whether different subgroups achieve different dietary guidelines (in the USA) (38). Metzgar et al. (39) did the same for a Paleolithic diet on a limited budget. Many consumers with a limited income have a low budget to buy food. This paper used the USDA data sets of the TFP. The results show that a Paleolithic diet is possible within the defined constraints. Nevertheless, the diet is too low in calcium and some other micronutrients. A 9.3% increase in spending was needed (39).

Darmon et al. (40) went into greater depth, testing the compatibility between nutrient profiling and recommendations based on nutrients by using LP. The option of modeling diets satisfying 40 nutrient constraints (“healthy models”) was tested. Healthy diets could be modeled using foods from the most favorable nutrient profile class, but unhealthy diets could not be modeled within an accurate scope of energy consumption. Darmon's conclusion was that a “few key nutrients (protein, fiber, saturated fatty acids, added sugars, sodium, vitamin C, calcium, iron) can be used to predict the ability of a given food to facilitate—or to impair—a large number of nutrient recommendations” (40).

In line with Darmon, Clervieulle assessed the validity of five different European nutrient profiling systems (i.e., Choices, Keyhole, French Agency for Food, Environmental and Occupational Health and Safety (AFSSA), European Commission (EC) system, and FoodProfiler). For each profiling system, construct validity was assessed by testing whether unhealthy foods (identified as non-eligible) results in unhealthy diets and healthy foods (those identified as eligible by the system) results in healthy diets. The AFSSA, EC, and FoodProfiler systems were proved to be valid, though some food products appeared to be misclassified. The two other systems failed. One important result was that, “it was possible to design healthy diets with eligible products and unhealthy diets with non-eligible products” (41).

The huge efforts in France resulted in the use of LP to develop dietary reference intakes as early as 2001 (42). Several studies have been conducted using data from a French representative dietary survey (ASPCC survey). All these studies have similar results and demonstrate that meeting nutritional requirements is difficult. But it is possible by using regular foods, by applying common nutritional recommendations (42, 43). Thus far, most studies using LP on diets focus exclusively on nutritional constraints (38, 39, 44–46). The studies of Ferguson et al. (44, 45) for example, were done to revise Food-Based Dietary Guidelines or to upgrade Food Aid. LP could be helpful to support development of dietary guidelines that fulfill all nutritional requirements.

### Economic constraints: food aid

Twenty of the studies included used economic constraints, five of them where focussed on food aid, 15 on applications in developed countries, of which three especially on the Thrifty Food Plan and two of them included also ecological constraints. The Diet Problem was originally designed to find low costs solutions for feeding soldiers (10). LP can also be applied to identify the lowest cost nutritionally adequate diet when providing food aid, as costs and nutrient content of foods are linearly associated to the weight of foods (11). Mathematical optimization models have long indicated (7, 8) that diets high in nutrients could be found very cheap (47).

LP was found to be very helpful in food aid programs. For example, Ryan et al. designed an LP tool to compose “novel ready-to-use therapeutic food” for malnourished children. They systematically surveyed international and national crop and food databases and took the example of ingredients locally available in Ethiopia. The cost of the optimized formulation was only $0.12 per 100 g, more than 40% cheaper than the available ready-to-use therapeutic food (48). Dibari et al. also published LP solutions for East Africa (49). LP can also be applied to calculate the lowest price for an additional food supplement necessary to reach an adequate diet. It can similarly be used to calculate what families save compared to the expenses by the donor after distributing a food supplement, for example in rural Chad (11). The same group stated that LP can help during the complementary feeding period. In a review article, Briend et al. informed pediatricians and public health professionals about this tool (3). Ferguson et al. addressed the diets of young children living in disadvantaged environments, for instance in Indonesia. They combined LP with goal programming. The study resulted in a number of optimal CFRs for the local population, giving insights in their most important “problem nutrients” (45).

From food aid in Africa, the next step was to apply LP to poor families and food banks in developed countries. Earlier, Briend and Darmon described an approach based on cost minimization by LP (in Excel) to determine which nutrients may be below recommended intakes in poor families in France (50). In 2007 Rambeloson et al. started a study on food banks in France, to assess the nutritional quality of the food distributed and to identify applicable changes to improve it. All 2004 data were collected for food aid donated by French food banks. LP was used to find the minimum changes necessary to meet the French dietary recommendations. The actual donation was improved by adding new foods into the food aid boxes (46). These examples demonstrate that cheap food is not always nutritionally adequate food. This has also been found in studies which only applied cost constraints.

### Economic constraints: costs vs. nutrients in developed countries

Although food aid was an important application of LP, the approach is not only useful for poor countries and disadvantaged citizens. Briend continued his work on LP in France, together with Darmon and Ferguson. Their focus was on the interactions between economic constraints and unhealthy diets. The study demonstrated that adding a cost constraint could result in diets with lower nutrient densities, with preferences comparable to the diets of low socioeconomic groups (51). This suggests that, when cost constraints affect food choices, LP resulted in an energy dense diet to maintain French dietary patterns (52). Next, Darmon et al. used LP to calculate the effect of a cost constraint on the available food choices for French women, to reach a healthy diet (53). Drewnowski and Spencer also found that reducing the costs of diets in LP models “leads to high-fat, energy-dense diets that are similar in composition to those consumed by low-income groups. Such diets are more affordable than more healthy diets based on lean meats, fish, fresh vegetables, and fruit” (54).

In 2007, Matthieu Maillot joined the French research team with new publications on cost constraints. For the first time, LP was applied on a large dataset of people, in this case a representative sample of French adults in the INCA data set (1999, *n* = 1,332) (55). Maillot et al. developed LP models to propose diets that satisfied higher nutritional constraints at minimum price. Their found agreement between LP and nutrient profiling indicates that “LP is a useful tool for testing nutrient profiling systems and validating the concept” (56). The work of Maillot et al. also concluded that “calculating the minimum cost of a nutritious diet needs to take social and cultural factors into account” (47). One of the studies performed in France also looked at the costs and found a 55% increase in costs for the nutritionally optimal diet, from € 2.75 to € 4.24 per 2,000 kcal (46). Similar work done by Maillot and Drewnowski in the US (2010) on optimizing the size of the servings and energy density also showed an increase in costs (to between $ 4.40 and $ 5.50/day, an increase of $ 0.10–1.20) (57).

Finally, LP with cost constraints has been applied to specific dietary requirements. Raffensperger (58) used LP to study the lowest available cost of a low-carbohydrate diet in New Zealand. Introducing constraints for carbohydrate and fat, resulted in a big, non-linear increase of cost. The study identified, within a low-carbohydrate, low-fat diet, which nutrients had the biggest effect on cost: The optimum diet cost NZ$ 14/day, with energy, calcium, and fiber being the most expensive nutrients (58). LP demonstrated to be an applicable tool to rigorously convert precise nutrient constraints into food combinations.

### The example of the thrifty food plan (TFP)

Of the 20 studies with economic constraints, 3 used the Thrifty Food Plan as an example. Forty years ago, the USDA developed the TFP to solve the problem of selecting a healthy diet for low-income groups. This dietary optimization program composed diets that fits within the constraints, using the 4,800 most popular foods. Since 1975, the TFP has been the most successful program providing healthful and minimal-cost meal plans and market baskets for consumers with a limited budget: more than 28 million. The TFP was updated in 1983, 1999, and 2006 (29). The researchers used 15 nutritional constraints (essential nutrients with official RDAs). In 1999, Lino et al. found that a family of four spent 23% of their income on unhealthy foods. In contrast with other studies, it was possible to increase the healthy components without changing the budget (29). USDA met simultaneously food group constraints, a cost constraint, and other constraints (30). The 2006 revision of the market baskets could meet food intake recommendations of the MyPyramid Food Guidance System (e.g., for fruit, vegetables, and milk). However, none of the market baskets was able to meet the sodium guideline, so sodium was limited for each age-gender group (59).

Wilde and Llobrera evaluated the TFP framework using constraints on food groups (e.g., meat, vegetables) or nutrients (e.g., saturated fat, calcium). It was possible to find nutritious diets for adult women with the TFP budget of $ 4.98/day, but it required a substantial change from current diets, or using nutrition standards in place of food category standards based on MyPyramid. This paper is interesting for future applications, because the authors introduced a stepwise approach, with the cost constraint increasing in steps of $0.05 (30). This stepwise approach is also applicable for other constraints and recently applied by Kramer et al. (12).

### Twelve studies with ecological constraints

The next step in the application of LP was the introduction of ecological constraints. Several studies—for instance in UK, and New Zealand—have successfully applied LP to optimize diets (4, 19, 33, 38, 39, 44, 46, 47, 57). This section gives an overview of the 12 studies which have applied ecological constraints to 14 diets between 2000 and 2016 (13, 16–26). The studies are summarized in Table 1.

Macdiarmid et al. were the first to use greenhouse gas emissions (GHGEs) as constraint. They found a realistic diet that could produce a 25 to 36% decrease in GHGEs (17, 33). Their study suggested that future work would need to integrate wider issues of sustainability into the modeling process and develop broader dietary advice (33). In the same period, Vieux et al. (60) designed a low-impact diet using not LP but a scenario, with 12% lower GHGEs (3.60 kg CO_2_eq/day) (60). The studies of Macdiarmid et al. (17, 33) and Vieux et al. (60) have drawbacks, as their diets include a low number of foods (82, 73 respectively]. The study by Van Dooren (20) included 206 most consumed Dutch food products, which is more realistic. It looked not only at GHGEs as an environmental parameter, but also at land use, energy use and ReCiPe score, which combines the three other parameters in an overall ecological pressure score. Later, Vieux et al. (61) improved and expanded the French dataset to 391 products. He did not use LP, but looked for associations between GHGEs and nutritional quality (61).

Macdiarmid et al.'s work modeling diets using LP methodology was supported by WWF in the United Kingdom with the goal of optimizing the nutritional quality of recommended diets and simultaneously reducing the diet-related GHGEs (17, 33). This method was also applied by WWF to program the national diets of France, Spain, and Sweden, using local available datasets and nutritional constraints (18, 62). The same kind of modeling was applied in a New Zealand study (19). Diets were first modeled without acceptability constraints. Then they applied diets with popular foods consumed by the population, with realistic amounts as constraints. They also applied constraints for food costs, energy intake, macronutrients, and micronutrients (9–11, 15, 17, 20, 26–36, 38–40) from national dietary recommended intakes. The optimized UK diets could result in 90% reductions in GHGEs, but the diet included only 7 foods and no drinks (and without emissions of the consumption phase). But this diet was without acceptability constraints (see section The Need for Acceptability Constraints) (33). The New Zealand study demonstrated similar reductions in GHGEs (19). The introduction of acceptability constraints in the UK model resulted in a diet incorporating 52 of the 82 typical food groups, without removing the groups meat or dairy, that met dietary recommendations and a 36% GHGEs reduction. The cost to the consumer did not increase. The modeled diets in France, Spain, and Sweden demonstrated similar results (18). The New Zealand study optimized 16 diets for nutritional adequacy, cost, and GHGEs (19). The latter study was of limited practical value, because the diets only include 14 to 19 foods, and drinks were not considered. The two studies of Van Dooren (20, 26) are more extensive, because they used 33 nutrients instead of 16 (17) or 18 (19). In conclusion, LP makes it possible to propose diets with lower impacts than diet scenario studies.

Based on nutrition recommendations, Donati et al. identified three different 7-day diets for the healthy Italian adult population (young adults from a high school in Parma, *n* = 104), characterized by different targets and optimizing different impacts: first the lowest cost diet (Minimum Cost Diet), then the Environmentally Sustainable Diet obtained by reducing the three environmental indicators (GHGEs, water consumption, and land use). Finally, the Sustainable Diet was recognized to reach simultaneously environmental and cost constraints. Donati et al. used 544 food items, but only 9 nutritional constraints (25). The added value of the study is the use of multiple environmental parameters and new acceptability constraints. They created a new constraint modeling the connection between matching food groups (biscuits as a complement with coffee or tea). To make the results more accurate, they argued that “at the same time, it is unlikely for some foods to be eaten during the same meal. For instance, it is not usual to eat both beef and fish. In modeling consumption behavior to reflect real world eating habits, the model incorporates an ‘alternative’ constraint avoiding the combination of certain food items in the same meal” (25). Despite these acceptability constraints, the volume of the diets almost doubled.

### Combining economic and ecological constraints

Nelson et al. (63) stated that: “There is limited and inconsistent evidence as to whether sustainable diets are more or less expensive than average diets” (63). Some LP studies added evidence.

The 2013 New Zealand study was the first study applying three types of constraints: nutritional, economic and ecological (GHGEs). The result was a monotonous diet containing 10 to 19 foods (selected from a database of 76 foods) (19). This study showed that the more food products and the higher the acceptability, the more expensive the diet was. Auestad and Fulgoni reviewed the results: “When the modeled diets included meals more familiar to New Zealanders, the cost tended to be higher than for the other optimized diets” (64). Concluding that: “Future research using this or similar approaches should also consider other aspects of environmental impacts (e.g., land use, water quality, food waste, and biodiversity), supply and pricing, including subsidies for farmers, and other social and economic aspects of sustainability” (64).

Van Dooren et al. (20) confirmed that costs increase when nutritional constraints are exclusively used. In contrast, adding environmental constraints decreased costs (20). In the second step of Van Dooren et al.'s optimization, they found that costs were not increased compared to the current Dutch diet, but reduced to € 3.20. This is substantially lower than the cost for the British Livewell 2020 diet (33): £ 29 per person per week (about € 4.80 a day), based on mid-range supermarket prices in August 2010. This may be due to differences in price levels, or differences in dietary patterns between the two countries. While € 3.20 is at the same price level as that found in a French study (4), that study excluded drinks and did not include GHGEs as a constraint. The optimized French diets are monotonous and are expected to have a low social acceptability. Lastly, a New Zealand study resulted in a cheaper diet (about € 1.92–2.44) (19). Though, these cheaper diets consist of low quantities of fruits and vegetables.

### Possibilities with quadratic programming

Although “Linear programming” was the selection criterium for this review, several selected diet studies were also using QP (31, 32, 51, 65). For example, Jalava et al. (13) assessed water footprints of diets after stepwise optimization (blue and green water, Table 1). They used QP to calculate stepwise the changes in diet gradually limit the percentage of animal protein to 50, 25, 12.5, and 0 of the total protein consumption of worldwide regions. Although QP is an optimization method (14), the goal was to find a diet that encounter the dietary guidelines per scenario with the lowest number of changes in the menu (retaining the typical diet for each country). The four applied scenarios resulted in reductions for the blue water footprint of 4, 6, 9, and 14. The original diet was assigned as the optimization objective. QP resulted in estimated cost for any scenario. Therefore, the result was close to the traditional, culturally acceptable diet and fulfilled the nutritional constraints (13). QP has advantages over LP when the goal is to find small changes on population level.

## Discussion

### Combining more constraints

Most studies have used nutritional constraints and cost constraints in the analysis of dietary problems and solutions, but such research begin showing weaknesses under situations featuring a small number of food items and/or nutritional constraints. The number of nutritional constraints vary from 5 to 37, which could have a major impact on the results of the studies: the lesser the number of constraints, the higher the risk of inadequacy of nutrient intake of the nutrients not considered. Even with a high number of nutritional constraints, bioavailability of nutrients (e.g., iron, amino acids) and phytochemicals are not considered. This could partly be solved by adding constraints on certain food groups rich in phytochemicals, e.g., fruits, vegetables, and fish. Future possibilities lie in finding LP solutions for diets by combining nutritional, costs, ecological and acceptability constraints. Evaluating the limited number of studies using LP on diets, we conclude that the studies of Wilson et al. (19) and van Dooren et al. (20) were unique in combining three dimensions: nutrients, GHGEs, and costs. Future LP diet studies should combine all three of these constraints.

### Comparability of ecological studies

The most important challenge to improve future LP diet studies with ecological constraints, is to build bigger databases with more foods and more environmental data, with improved quality and consistency of the data.

Although the papers cited above (33, 66) observed substantial reductions in GHGEs, it is striking that they found much higher emission levels—in absolute terms—than the Dutch study (20). The found emission of 1.56 kg CO_2_eq/day is lesser than the 3.6–4.2 kg for France and 3.77–5.02 kg for the UK (Macdiarmid also calculated a very limited diet with a 70% reduction in GHGEs, resulting in 1.74 kg CO_2_eq, which is still higher than the Dutch results). On the other hand, one of Wilson et al.'s results in New Zealand is comparable: 1.62 kg CO_2_eq, but without drinks (19). These differences may be explained by different methods used to calculate GHGEs per product or variances in food cultures and preferences. This approves the preferability of a “country-by-country approach” (20); outcomes should not be extrapolated from one country to another, because of the differences in availability of reliable data, dietary patterns, and the climate impact of products (20).

Table 2 makes it clear that the studies differ in number of food items (13–544; an indication of the completeness of diets), the size of the population, the number of nutritional constraints (5–33; an indication of the nutritional quality of the diets), the selected economic and ecological constraints, and the solutions to make the outcomes culturally acceptable.

**Table 2:** Twelve diet studies with nutritional and ecological constraints.

**Study**	**Country/region**	**Individual/population**	**Diet of**	**No. of food items**	**No. of food groups**	**Programme**	**Optimization on (objective function)**	**Nutritional constraints**	**Economic constraints**	**Ecological constraints**	**Other parameters calculated**	**Acceptability constraints**
(13)	176 countries	Pop.	National food supply FAO		13	? Quadratic	Minimize change in diets	5	x	Blue and Green Water	Overeating + food deficiency	No change in fish, spices, and stimulants; no increase of alcohol and sugar; stepwise decrease of animal protein: 50, 25, 12.5, 0%.
(16)	England and Wales	Pop.	Households		167	? Quadratic. See Srinivasan et al. (67)	Minimize changes % in diet, + expenditure changes	13	x	Land Use	Cost of labor	
(17)	United Kingdom		Women, 19–50 y	52/82		GNU kit implemented in Rglpk of R statistical software	GHGE (?)	16	British pounds	GHGEs		
(18)	Spain	Pop.		277		Rglpk package	Minimum GHGE (> 25% reduction)	17	Only as outcome	GHGEs	Costs	Amounts consumed in particular food groups > 60–80% of the current average consumption
	Sweden	Pop.		88		Rglpk package	Minimum GHGE (> 25% reduction)	21	Only as outcome	GHGEs	Costs	
	France	Pop.		68	13	Solver in Excel	> 25% reduction GHGE	13	Only as outcome	GHGEs	Costs	On particular portion sizes for each food and minimal departure from the average diet
(19)	New Zealand		Males, 16 diets	76	14–18	Excel, R language	Nutritional requirements (?)	17	NZ$	GHGEs	Food waste UK	
(20)	NL		Males and females 31–50 y	206		Optimeal (Matlab)	Popularity (kg)	33	Euros	GHGEs	Land use + ReCiPe score	
(21)	NL	Ind.	Females 31–50 y	207		Optimeal (Matlab)	Penalty score on popularity (kg)	37	x	pReCiPe	GHGE + Land Use + Energy Use	Penalty score <100; no constraints on food groups
(22)	UK	Pop.	Adult males and females		42 (148 sub)	Software R 2012, package Alabama. Nonlinear with Augmented Langrangian method.	Squared deviations in “loss of welfare” from current diet	14	x	GHGEs		Stepwise reduction 10–70%; max. 50% deviation is acceptable; loss of welfare: expenditure shares/own-price elasticities
(23)	France	Pop.	French INCA2 dietary survey, adults *n* = 1,899	402	8	Statistical software package SAS version 9.4	Minimizing the total departure between the diets at food item and group level	33	Only as outcome	GHGEs	Mean adequacy ratio; Mean excess ratio; Solid energy density	Total weight (80–120%), <90th percentile for foods and food groups
(24)	UK	Pop.	UK National Diet and Nutrition Survey, adults *n* = 1491		134	GNU Linear Programming Kit implemented in IpSolveAPI package of R stat software.	Minimizing the changes to their current intake	27	x	GHGEs (−25%)		1. gradual changes (≤50%) to amount of any foods currently eaten. 2. New foods were added. 3. Greater reductions (≤75%). 4. foods were removed
(25)	Italy, Parma	Sub-pop.	Young adults (18–20y) high school *n* = 104	544		? Multi-Objective Linear Programming	Minimizes both consumer expenditure and environmental impact	9	Euro	GHGEs, Land Use, water footprint	Simultaneously 3	1. Food portion, 2. Food consumption frequency, 3. Food association, 4. Food alternative
(26)	NL	Pop.	Male adults (31–50 y), historical	206		Optimeal (Matlab)	Popularity (kg)	33	x	GHGEs, Land Use combined	Distance in Health Score; No. of products added, eliminated and changed	Popularity (normalized value of the total food consumption based on weight)

*The table gives details about the goal, objective function, selected population group, program, number of food items, and outcomes*.

### The need for acceptability constraints

One of the attempts to make outcomes culturally acceptable, is the introduction of acceptability constraints. Six studies demonstrated good examples of those constraints. From the first studies of Dantzig to date, researchers have struggled with the unrealistic outcomes of LP solutions. It was expected that adding acceptability constraints could help to prevent this. A good example is Maillot et al.'s (43) study, whose objective was “to assess the feasibility of achieving a set of 30 nutrient recommendations at the individual level and to characterize factors associated with feasibility.” The diets of all participants the French national food consumption survey (*n* = 1,171) were optimized. For everyone, departure from his/her recorded diet was minimized:

- Only foods reported in his/her weekly diet record were used to fulfill a set of nutritional constraints.
- Acceptability constraints guaranteed warranted accurate portions and patterns. For any given food, upper limit on the quantity was defined by the 95th percentile of consumer intake.
- To keep away from unacceptable quantities of food, the optimized diet should be lower than 115% of the weight eaten per week.
- Model feasibility was calculated for all diets.

The vitamin D constraint was the most difficult to fulfill, followed by sodium, magnesium, and saturated fatty acids. The new approach resulted in a “method for identifying nutrient levels that need to be carefully evaluated when establishing recommendations” (43).

However, the use of cultural acceptability constraints limits finding solutions. In 2016 Parlesak et al. collected average prices for 312 foods available within Copenhagen, Denmark. They calculated five different cost-minimized food baskets for a family of four. The food baskets that met food based dietary guidelines was twice the price. Introducing cultural acceptability constraints increased the cost three times. So, variety in the diet and cultural acceptability has a price (68).

Thompson et al. (18) also struggled with the issue of acceptability and used a lot of trial and error. In their study at least 30% of the most popular foods were retained. They also put an upper bound on most foods and removed foods with smaller amounts in the diet, as well as less healthy options such as full-fat milk. They applied lower bounds of consumption, particularly on popular foods. For example, bread, potatoes and pasta have comparable GHGEs and prices, but the model will try to optimize one of the products for cultural reasons: for instance, consumption of potatoes was limited in Spain and pasta in Sweden (18).

Other examples of the improvement of LP methodology were demonstrated in literature by using more nutritional constraints (47) and selecting most frequently consumed foods (4, 69). Maillot et al. improved LP models by using a goal function to maximize most frequently consumed foods, without replacing more than five products from the current diet (4). Van Dooren et al. (20) implemented this by using a unique objective function, maximizing the most consumed food products based on weight and minimizing absolute change in portions. For example, in the men's diet 50 of the 83 products were kept unchanged in number of calculated portions, and in the women's 55 of the 73 products. The Optimeal tool calculated a change in portions for 8 foods for men and 7 for women. Finally, 9 new food items were added to the men's diet and 8 to the women's (unsalted peanuts, pear, kale, sauerkraut, lentils, marrowfats, soy drink, mackerel, and mussels). Nevertheless, the diet was almost vegetarian, with less portions of meat and dairy. Likewise, new products such as soy drink, marrowfats and lentils were added to the diet, which are not consumed by the majority of the Dutch population (20). A reality check is needed to determine if this would be acceptable for consumers.

**Figure 3:**
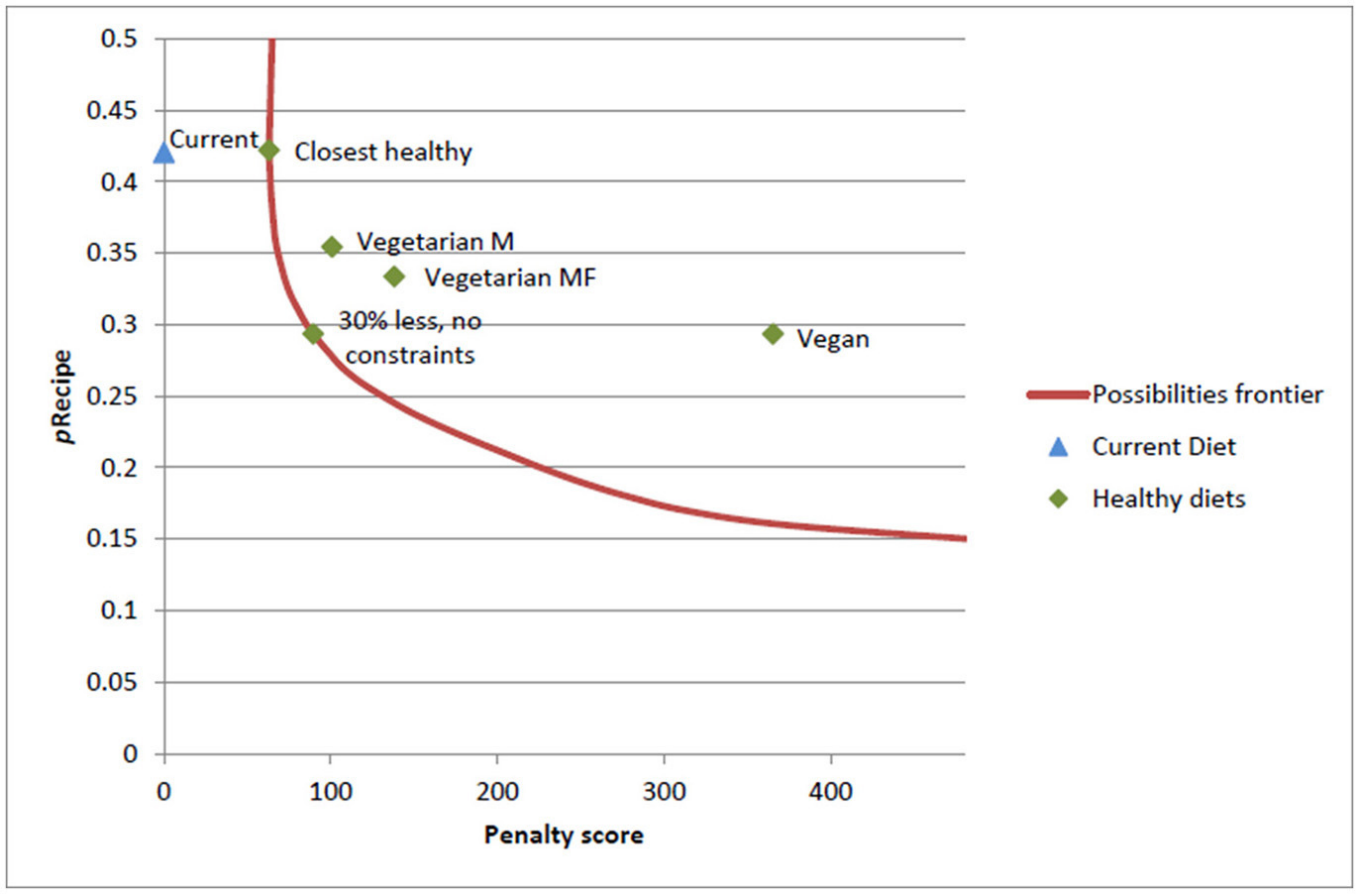
Example of the application of acceptability constraints and the effects on the environmental impact of different diet scenarios (M, males; F, females). The lower the penalty score is, the closer the diet is to the current diet and the more acceptable (21). The red line is called the “possibilities frontier.” It indicates the possibilities with the lowest penalty score for a certain environmental constraint (21).

Tyszler et al. (21) described the application of a penalty score as acceptability constraint in more detail. The metric for changes was measured by a penalty score based on the popularity of foods. More specifically, “the number of servings changed in each food is multiplied by a normalization of the total quantity of that food (grams) previously consumed” according to the dietary survey (21). Tyszler et al. explained: “The penalty score can be interpreted as a measure of distance between diets. The reasoning behind this modeling is that diets which are like the current one is more likely to be accepted by most of the population than more extreme diets.” The results of their study are shown in Figure 3: “the closer a diet is to the frontier line, the more similar it is to the current diet, while being healthy” (21). The “Closest healthy” and the “30% less” environmental impact diet (expressed as Recipe-scores; Recipe is a unit used in LCA methods) are, by definition, on the frontier. The figure indicates that, if the goal of the optimization is a diet with lower environmental load Vegetarian or Vegan are not the only options. There are many other solutions to this diet problem with a smaller number of adaptations in the diet (21). Introducing acceptability constraints is recommended, but no study has provided the ultimate solution to calculating acceptability.

### Proven value of LP in diet studies

Although the Diet Problem has a long history, most diet solutions are from 2000 or later, as computers with larger calculation capacity became widely available and LP tools were developed. The literature shows that LP can be applied to a variety of diet problems: from food aid, national food programs, dietary guidelines, to individual solutions. In supporting dietary guidelines, LP has proven its value in many ways. Most studies have used nutritional constraints combined with cost constraints. Studies showed weaknesses when the number of food items and/or nutritional constraints were low. However, even when the number of constraints is increased, LP is not always able to find solutions. Nutritional constraints should reflect at least the national dietary guidelines. In defining affordable diets and investigating the relationship between cost and health, LP studies provided insightful contradictions. LP shows that cheaper and healthier foods can be found easily, but when price becomes a constraint, often a shift occurs to unusual food unless the right constraints are chosen. LP can produce solutions that are not realistic for the population, especially when cultural acceptability is not considered. Introducing acceptability constraints is recommended, but none of the studies provide the ultimate solution for calculating acceptability. LP can play a role in the future developments on acceptance of changes and personalized food.

### Choice of function and tool

Table 2 demonstrated that the analyzed studies are not always clear about the choice of their programming tool and objective function. Arnould et al. (16) and Javala et al. (13) seems to apply QP, but that is not even clear. Macdiarmid et al. (17) and Wilson et al. (19) did not describe where the optimization is based on (GHGEs or nutritional requirements?). It should be expected that the methods are clearly described. The older software tools (Rglpk package, R stat software and Solver in Excel) are still in use and seem to function well, but because of the complexity of the diet problem, more sophisticated and tailor-made tools are built for specific application (Optimeal and Cost of the Diet-tool). Further development is needed to implement acceptability constraints.

Quadratic Programming has many advantages over LP when you want small changes on population level. QP differs from LP in that the functions are not linear but quadratic. An inherent limitation of LP is that it limits the amount of changes, while sometimes a wider range of small changes in products can give more useful solutions, e.g., when changing diets on population level. QP have this advantage above LP.

## Conclusions

LP could be helpful to support development of dietary guidelines that fulfill all nutritional requirements. LP also demonstrated to be an applicable tool to conscientiously convert predefined nutrient constraints into diets with unpredictable food combinations. Most studies have used nutritional constraints and cost constraints in the analysis of dietary problems and solutions, but such research begin showing weaknesses under situations featuring a small number of food items and/or nutritional constraints. Introducing acceptability constraints is recommended, but no study has provided the ultimate solution to calculating acceptability. Only 12 studies applied and introduced ecological constraints (and of these, only two also included cost constraints). These studies showed that the environmental impacts of diets can be halved, staying within the existing nutritional constraints. LP makes it possible to propose diets with lower impacts than diet scenario studies. In other words, LP is an important tool for environmental optimization and has a lot of potential. Important is consistency in methodology to derive environmental figures (full scope) and completeness of constraints. Future possibilities lie in finding LP solutions for diets by combining nutritional, cost, ecological, and acceptability constraints. LP is clearly a very helpful instrument for finding solutions to a variety of very complex diet problems.

## Author contributions

The author confirms being the sole contributor of this work and approved it for publication.

### Conflict of interest statement

The author declares that the research was conducted in the absence of any commercial or financial relationships that could be construed as a potential conflict of interest.

## Appendix

**TABLE A1:** Short overview of the reviewed papers, year published, type of programming (linear or quadratic) and constraints used.

**Reference**	**Year**	**LP or QP**	**Constraints**				**Comments**
			**Nutrients**	**Costs**	**Environment**	**Acceptability**	
50	2000	LP	N			A	A corresponds to portion size
11	2001	LP	N			A	A corresponds to portion size
29	2001	LP	N			A	A corresponds to portion size
42	2001	LP	N				
34	2002	LP	N			A	A corresponds to portion size
51	2002	QP	N	C		A	A corresponds to portion size
3	2003	Review					
52	2003	LP	N	C		A	A corresponds to portion size
65	2003	QP	N			A	A corresponds to portion size
44	2004	LP	N			A	A corresponds to portion size
54	2004	LP	N	C			
31	2006	QP	N			A	A corresponds to portion size
45	2006	LP	N	C		A	A corresponds to portion size
53	2006	LP	N	C			
55	2007	LP					It does not perform optimization
59	2007	LP	N	C		A	A corresponds to portion size and the amount to be consumed
32	2008	QP	N				
46	2008	LP	N	C			
56	2008	LP	N			A	The author explicitly states that it includes nutrient (N) and acceptability restrictions (A)
58	2008	LP	N				
30	2009	LP	N	C			
35	2009	LP	N	C		A	A corresponds to the deviation from current consumption. And at least one of the 4 models developed includes cost constraints (C)
38	2009	LP	N				
40	2009	LP	N			A	A corresponds to portion size
43	2009	LP	N			A	
4	2009	LP	N			A	
47	2010	LP	N				
16	2010	LP	N		E		
33	2010	LP	N	C			
39	2011	LP	N	C			The author explicitly states that it includes cost restrictions (C)
57	2011	LP	N			A	A corresponds to the deviation from current consumption
69	2011	LP	N				It does not include acceptability in the restrictions
17	2012	LP	N				
36	2012	LP	N			A	A corresponds to portion size
49	2012	LP	N	C			
41	2013	LP	N				
19	2013	LP	N	C	E		
18	2013	LP					It was not possible to access
9	2014	LP	N			A	A corresponds to the deviation from current consumption
48	2014	LP	N				It does not include cost restrictions; it mentions that it can be included in the developed tool
13	2014	QP	N			A	A corresponds to the deviation from current consumption
20	2015	LP	N	C	E	A	A corresponds to the deviation from current consumption
22	2015	LP	N		E		
15	2016	Review					
26	2016	LP	N		E	A	A corresponds to the deviation from current consumption
12	2016	LP	N		E	A	A corresponds to the deviation from current consumption
23	2016	LP	N		E		
25	2016	LP	N		E	A	A corresponds to the deviation from current consumption
24	2016	LP	N		E		
68	2016	LP	N			A	Costs included in the objective function, not in the restrictions
21	2016	LP	N		E	A	
