# Corrigendum: A Review of the Use of Linear Programming to Optimize Diets, Nutritiously, Economically and Environmentally

**DOI:** 10.3389/fnut.2022.850033

**Published:** 2022-05-13

**Authors:** Javiera García-Leal, Andrea Teresa Espinoza Pérez, Corné van Dooren, Óscar C. Vásquez

**Affiliations:** ^1^Program for the Development of Sustainable Production Systems (PDSPS), Faculty of Engineering, University of Santiago de Chile (USACH), Santiago, Chile; ^2^Industrial Engineering Department, Faculty of Engineering, University of Santiago de Chile (USACH), Santiago, Chile; ^3^Voedingscentrum, The Netherlands Nutrition Centre, Den Haag, Netherlands

**Keywords:** sustainable diet, linear programming, diet costs, nutritional quality, environmental constraints (EC)

In the original article, there was a mistake in Table A1 as published. Reference [41] was duplicated twice in the table. Additionally, there were errors in the classification of the articles presented in Table A1. In the Acceptability column the original paper did not consider “portion size” as a constraint to diet optimization. In the revised Table A1 this has been added in the column Comments. The corrected Table A1 appears below. Full reference details for the references cited in the table can be found in the original article.

**TABLE A1:** Short overview of the reviewed papers, year published, type of programming (linear or quadratic) and constraints used.

**Reference**	**Year**	**LP or QP**	**Constraints**				**Comments**
			**Nutrients**	**Costs**	**Environment**	**Acceptability**	
50	2000	LP	N			A	A corresponds to portion size
11	2001	LP	N			A	A corresponds to portion size
29	2001	LP	N			A	A corresponds to portion size
42	2001	LP	N				
34	2002	LP	N			A	A corresponds to portion size
51	2002	QP	N	C		A	A corresponds to portion size
3	2003	Review					
52	2003	LP	N	C		A	A corresponds to portion size
65	2003	QP	N			A	A corresponds to portion size
44	2004	LP	N			A	A corresponds to portion size
54	2004	LP	N	C			
31	2006	QP	N			A	A corresponds to portion size
45	2006	LP	N	C		A	A corresponds to portion size
53	2006	LP	N	C			
55	2007	LP					It does not perform optimization
59	2007	LP	N	C		A	A corresponds to portion size and the amount to be consumed
32	2008	QP	N				
46	2008	LP	N	C			
56	2008	LP	N			A	The author explicitly states that it includes nutrient (N) and acceptability restrictions (A)
58	2008	LP	N				
30	2009	LP	N	C			
35	2009	LP	N	C		A	A corresponds to the deviation from current consumption. And at least one of the 4 models developed includes cost constraints (C)
38	2009	LP	N				
40	2009	LP	N			A	A corresponds to portion size
43	2009	LP	N			A	
4	2009	LP	N			A	
47	2010	LP	N				
16	2010	LP	N		E		
33	2010	LP	N	C			
39	2011	LP	N	C			The author explicitly states that it includes cost restrictions (C)
57	2011	LP	N			A	A corresponds to the deviation from current consumption
69	2011	LP	N				It does not include acceptability in the restrictions
17	2012	LP	N				
36	2012	LP	N			A	A corresponds to portion size
49	2012	LP	N	C			
41	2013	LP	N				
19	2013	LP	N	C	E		
18	2013	LP					It was not possible to access
9	2014	LP	N			A	A corresponds to the deviation from current consumption
48	2014	LP	N				It does not include cost restrictions; it mentions that it can be included in the developed tool
13	2014	QP	N			A	A corresponds to the deviation from current consumption
20	2015	LP	N	C	E	A	A corresponds to the deviation from current consumption
22	2015	LP	N		E		
15	2016	Review					
26	2016	LP	N		E	A	A corresponds to the deviation from current consumption
12	2016	LP	N		E	A	A corresponds to the deviation from current consumption
23	2016	LP	N		E		
25	2016	LP	N		E	A	A corresponds to the deviation from current consumption
24	2016	LP	N		E		
68	2016	LP	N			A	Costs included in the objective function, not in the restrictions
21	2016	LP	N		E	A	

In the original article there was also an error in reference [47]. The reference for [47] was incorrectly written as “Maillot M, Darmon N, Drewnowski A. Are he lowest-cost healthful food plans culturally and socially acceptable? *Public Health Nutr*. (2010) 13:1178–85. doi: 10.1017/S1368980009993028”. It should be “Maillot M, Darmon N, Drewnowski A. Are the lowest-cost healthful food plans culturally and socially acceptable? *Public Health Nutr*. (2010) 13:1178–85. doi: 10.1017/S1368980009993028”.

In the original article, there also was an error in the section Results, Twelve Studies With Ecological Constraints, Paragraph 1. In the list “France, UK, and New Zealand”, France should not have been included and the reference [60] should not have been included. The updated paragraph appears below.

Several studies—for instance in UK, and New Zealand—have successfully applied LP to optimize diets (4, 19, 33, 38, 39, 44, 46, 47, 57). This section gives an overview of the 12 studies which have applied ecological constraints to 14 diets between 2000 and 2016 (13, 16–26). The studies are summarized in Table 1.

The authors apologize for these errors and state that this does not change the scientific conclusions of the article in any way. The original article has been updated.

## Author Contributions

AE, JG-L, and ÓV contributed to the conception and the design of this corrigendum and performed the analytic review. JG-L organized the database and first references' analysis of this corrigendum and wrote the first corrigendum draft of the manuscript. ÓV, AE, and CD wrote sections of the manuscript. All authors contributed to manuscript revision, read, and approved the submitted version.

## Publisher's Note

All claims expressed in this article are solely those of the authors and do not necessarily represent those of their affiliated organizations, or those of the publisher, the editors and the reviewers. Any product that may be evaluated in this article, or claim that may be made by its manufacturer, is not guaranteed or endorsed by the publisher.

